# Persistent hyperparathyroidism and its predictive factors in end-stage renal disease patients after kidney transplantation: A prospective study

**DOI:** 10.1097/MD.0000000000051216

**Published:** 2026-10-16

**Authors:** Kim-Ngan Huynh Lam, Hen Huu Phan, Sam Minh Thai, Minh Duc Do

**Affiliations:** a Department of Physiology-Pathophysiology-Immunology-Pharmacology, School of Medicine, University of Medicine and Pharmacy at Ho Chi Minh City, Ho Chi Minh City, Vietnam; b Department of Endocrinology, Cho Ray hospital, Ho Chi Minh, Vietnam; c Department of Urology, Cho Ray hospital, Ho Chi Minh, Vietnam; d Center for Molecular Biomedicine, University of Medicine and Pharmacy at Ho Chi Minh City, Ho Chi Minh City, Vietnam.

**Keywords:** kidney transplant, persistent parathyroidism

## Abstract

Hyperparathyroidism (HPT) may persist in patients after kidney transplantation (KT). A better understanding of which patients are likely to have persistent HPT after KT would help in the development of optimal management strategies. Our aim was to examine the prevalence of persistent HPT after KT in a contemporary cohort and to identify risk factors associated with persistent HPT at 6 months. We followed 104 KT subjects in a longitudinal prospective cohort at Cho Ray Hospital, Vietnam, from October 2023 to May 2025. Persistent HPT was defined as a parathyroid hormone (PTH) level of 150 pg/mL at 6 months after KT. Among the 104 patients, the prevalence of persistent HPT at 6 months was 58.65%. The average PTH levels pretransplant and at 6 months posttransplant were 375.70 (IQR = 259.00–603.25) pg/mL and 167.95 (IQR = 112.53–227.30) pg/mL, respectively. Patients with persistent HPT had significantly higher baseline PTH (median 489 vs 273, *P* < .001) and alkaline phosphatase levels (median 102.0 vs 89.5, *P* = .025), and also experienced longer durations of chronic kidney disease (median 5.0 vs 2.0, *P* = .007) and dialysis therapy (2.0 vs 1.0, *P* < .001). Higher pretransplant intact PTH was independently associated with persistent HPT across both multivariable models, whereas the association with dialysis duration was significant only when categorized as ≥1 year. Our findings suggest that high pretransplant intact PTH independently predicts persistent HPT at 6 months, whereas dialysis duration is only significant when it reaches 1 year or longer.

## 1. Introduction

Kidney transplantation (KT) is considered to be the gold standard for renal replacement therapy for patients with end-stage renal disease (ESRD). KT is very effective in correcting the endocrine and metabolic disorders associated with chronic kidney disease (CKD) immediately after transplantation.^[1]^ Although kidney function significantly improves following successful engraftment, hyperparathyroidism (HPT) often does not completely resolve, a phenomenon known as persistent HPT (or posttransplant HPT).^[2,3]^ These findings indicate that enhanced renal function does not necessarily result in complete recovery from HPT, pointing to a complex underlying mechanism.

The reported prevalence of persistent HPT after KT varies widely across studies, ranging from 50% to over 80% of kidney recipients after 1 year of transplantation.^[4,5]^ These significant differences can be explained by various factors, including the type of parathyroid hormone (PTH) assay used, the specific PTH cutoff value chosen to define persistent HPT, the timing of the assessment, and the study design. Sustained elevated PTH levels posttransplant are associated with numerous adverse outcomes, which include bone mineral density loss, increased fracture rates, renal allograft dysfunction, and other transplant-related complications.^[6–8]^ With these severe complications, persistent HPT is not only a biochemical abnormality but is also a significant clinical burden affecting long-term outcomes for transplant recipients. Understanding and managing persistent HPT is therefore not just an endocrinological concern but a crucial factor in optimizing overall posttransplant care and ensuring the long-term success of KT.

Given the current debate about the optimal timing for treating persistent HPT following KT, a better understanding of the natural course of HPT and metabolic recovery is essential for mitigating adverse effects on transplant outcomes. Further, identifying factors associated with persistent HPT would help in devising appropriate monitoring strategies and improving patient prognosis. Therefore, this study aims to evaluate the natural course of HPT and to identify factors associated with persistent HPT after successful KT.

## 2. Methods

### 2.1. Study population and design

We performed a longitudinal prospective cohort study from October 2023 to May 2025 involving KT recipients admitted to Cho Ray Hospital, Ho Chi Minh City, Vietnam. All participants had to fulfill the following criteria: recipient from a living donor, first renal allograft, and no parathyroidectomy before KT. The criteria for exclusion consisted of the following: undergoing KT at a non-study center, having incomplete datasets, suffering from graft failure during the subsequent monitoring period, or being under the age of sixteen at the time of the operation. All participants provided written informed consent before enrollment. No formal a priori sample-size calculation was performed. This prospective observational study consecutively enrolled all eligible kidney transplant recipients during the predefined recruitment period.

Successful KT was defined as survival with a functioning kidney allograft, sustained independence from dialysis after the immediate posttransplant period, and absence of graft loss during the 6-month follow-up. Graft function was longitudinally assessed using serum creatinine and estimated glomerular filtration rate (eGFR) calculated using the CKD-EPI equation. We did not impose a fixed eGFR threshold because functioning kidney allografts may exhibit different levels of eGFR, and serum creatinine or eGFR alone does not comprehensively define graft success.

### 2.2. Participant recruitment

During the study period, a total of 142 kidney transplant recipients were screened for eligibility. Of these, 32 patients were excluded because pretransplant (iPTH) measurements were unavailable, as they had undergone KT at other centers. An additional 6 patients who received deceased-donor kidney transplants were excluded because the study was restricted to living-donor kidney transplant recipients. Consequently, 104 eligible patients were included in the study and completed the required posttransplant follow-up assessments.

Although most patients had been treated with either calcium or vitamin D derivatives at some point before their transplant, access to newer secondary hyperparathyroidism (SHPT) therapies like calcimimetics and non-calcium phosphate binders remained extremely limited. This was chiefly due to poor local availability, prohibitive pricing, and an absence of insurance reimbursement, which together restricted optimal management options for ESRD patients. Moreover, in the posttransplant phase, no individual in this group received calcium, vitamin D products, or any pharmaceuticals known to affect calcium metabolic pathways.

### 2.3. Data collection

Clinical and demographic characteristics, including age, gender, body mass index, etiology for ESRD, type of dialysis, and years of dialysis, were recorded. The following biochemical parameters were collected: ionized calcium, phosphate, 25OH-vitamin D, alkaline phosphatase (ALP), iPTH, serum creatinine, and eGFR using the CKD-EPI equation. The biochemical measurements were performed immediately before KT and at 1, 3, and 6 months after transplantation. The standard immunosuppression protocol for all patients comprised prednisolone, tacrolimus, and mycophenolate mofetil. The flow chart of the study is presented in Figure 1.

**Figure 1.**
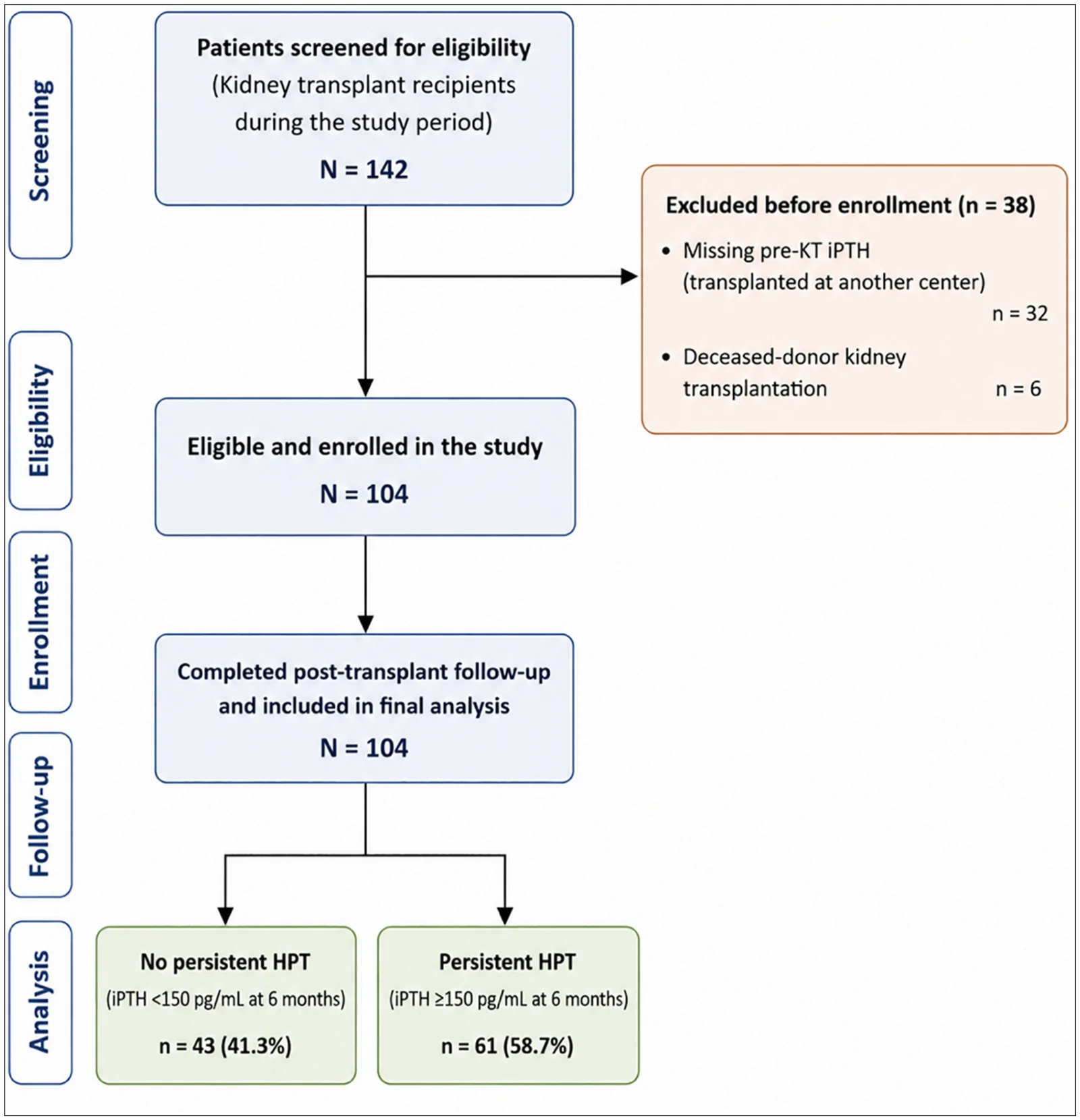
Flow chart of the study. HPT = hyperparathyroidism, iPTH = intact parathyroid hormone, KT = kidney transplantation.

### 2.4. Definitions of post-transplantation HPT

Currently, there is no consensus on a cutoff PTH level identifying persistent HPT. However, Araujo et al showed that an iPTH cutoff value of 150 pg/mL at 6 months after KT had the highest specificity for persistent HPT at 1 year.^[7]^ Hence, posttransplant persistent HPT was defined as PTH ≥ 150 pg/mL at 6 months postKT in this study.

### 2.5. Measurements

Plasma concentrations of creatinine, ionized calcium, phosphate, and ALP were measured at each study visit. Estimated eGFR was calculated using the CKD-EPI formulation. Serum iPTH levels were determined using a 2-site sandwich immunoassay (ARCHITECT i2000SR, Abbott Laboratories; reference range, 15–68.3 pg/mL). Serum 25-hydroxyvitamin D (25[OH]D) levels were determined using a direct competitive chemiluminescence immunoassay (LIAISON® 25 OH Vitamin D TOTAL Assay, DiaSorin, Italy). Serum ALP and phosphate levels were measured using an automated clinical chemistry analyzer (ADVIA® 1800 Chemistry System, Siemens Healthineers, Germany), with phosphate determined using the phosphomolybdate/UV method. Ionized calcium was measured using an ion-selective electrode method (RAPIDLab® 1265 Blood Gas System, Siemens Healthineers, Germany). Other routine biochemical parameters, including serum creatinine, were measured using an automated clinical chemistry analyzer (cobas® c 701 module, Roche Diagnostics, Germany).

### 2.6. Statistical analysis

The analyses were performed using SPSS computer software version 27.0 (IBM Corp). Descriptive analysis results for the variables were expressed as mean ± standard deviation, median (interquartile range), and count (percentages) for parametric, nonparametric distribution, and categorical variables, respectively. Independent *t* tests, Mann-Whitney tests, Chi-squared, and Fisher’s exact tests were used to examine the differences between the compared groups.

Longitudinal changes in biochemical parameters measured before transplantation and at 1, 3, and 6 months after transplantation were analyzed using linear mixed-effects models to account for within-patient correlation arising from repeated measurements. Time was modeled as a categorical fixed effect, and a patient-specific random intercept was included to account for between-patient heterogeneity. An unstructured covariance structure was used for repeated measurements. Overall longitudinal change was assessed using the fixed effect of time. When the overall time effect was statistically significant, post hoc pairwise comparisons between time points were performed using estimated marginal means with Bonferroni adjustment for multiple comparisons. Model assumptions were assessed by inspection of residual distributions and Q–Q plots. Variables with markedly right-skewed distributions were log-transformed before modeling when necessary. Mixed-effects models used all available observations under the missing-at-random assumption; therefore, participants with an occasional missing biochemical measurement were not excluded from the entire longitudinal analysis. Two-sided *P* values < .05 were considered statistically significant.

Univariate and multivariate logistic regression were used to investigate the causal relationship between explanatory variables and posttransplantation persistent HPT and to identify the associated factors for developing persistent HPT. A two-tailed *P* value < .05 was considered statistically significant. Candidate variables for multivariable analysis were selected a priori based on clinical relevance and evidence from previous studies, with additional consideration of their associations in univariable analyses. Variables associated with persistent HPT at *P* < .05 in univariable analysis were considered for inclusion. To limit model complexity and reduce the risk of overfitting, 4 predictor parameters were included in each multivariable model: CKD duration, dialysis duration, pretransplant iPTH, and pretransplant ALP. With 61 persistent-HPT events, this corresponded to approximately 15.3 outcome events per model parameter. Model 1 included these variables in their continuous form, whereas Model 2 categorized CKD duration, dialysis duration, and pretransplant iPTH according to the prespecified thresholds, with ALP retained as a continuous variable. Multicollinearity was assessed using variance inflation factors (VIFs) and tolerance statistics, with VIF < 5 and tolerance >0.20 considered indicative of the absence of problematic multicollinearity. Model fit was assessed using the Hosmer–Lemeshow goodness-of-fit test and Nagelkerke *R*^*2*^, and model discrimination was evaluated using the area under the receiver operating characteristic curve (AUC) with 95% confidence intervals. Effect estimates are presented as adjusted odds ratios (ORs) with 95% confidence intervals (CIs).

Missing data were assessed before statistical analysis. No missing data were present for the primary outcome (serum iPTH at 6 months after transplantation). Occasional missing values in secondary biochemical variables were infrequent (<5% for each variable) and were assumed to be missing completely at random. Therefore, no data imputation was performed, and complete-case analysis was used for analyses involving these variables.

## 3. Results

### 3.1. Baseline characteristics of the recipients and changes in biochemical parameters after KT

The 104 KT recipients had a median age of 35 at the time of KT. Of these patients, 56.7% were male, and the most common etiology of ESRD was unknown (68.3%), followed by glomerulonephritis (21%). The median duration of CKD was 3 years (IQR = 2.00–6.00). The majority of the cohort (93.3%) underwent hemodialysis pre-KT, with a median time on dialysis of 1 year (IQR = 0.85–2.00). All patients underwent living donor KT; 86 cases (82.7%) were ABO blood group compatible with their donor, and 71% achieved a complete HLA match. The median iPTH level was 375.70 pg/mL. The baseline characteristics of the cohort are described in Table 1.

**Table 1:** Characteristics of KT recipients at admission for KT by posttransplant HPT.

Characteristics	Total (n = 104)	HPT status 6 mo after KT		*P*-value
		No HPT (n = 43)	HPT (n = 61)	
Age at KT (yr) Median (IQR)	35.00 (31.00–41.00)	37.00 (31.00–40.50)	35.00 (30.00–41.00)	.550
Gender N (%)				.519
Female	45 (43.30%)	17 (39.50%)	28 (45.9%)	
Male	59 (56.70%)	26 (60.50%)	33 (54.1%)	
ABO compatibility with donor N (%)				.816
Compatibility	86 (82.70%)	36 (83.70%)	50 (82.00%)	
Incompatibility	18 (17.30%)	7 (16.30%)	11 (18.00%)	
Duration of CKD (yr) Median (IQR)	3.00 (2.00–6.00)	2.00 (2.00–3.00)	5.00 (2.00–7.00)	.007
Cause of ESRD N (%)				.277
Glomerulonephritis	21 (20.20%)	7 (16.28%)	14 (22.95%)	
Hypertension	12 (11.50%)	3 (6.98%)	9 (14.75%)	
Other/ Unknown	71 (68.30%)	33 (76.74%)	38 (62.30%)	
Type of dialysis N (%)				.300
None	6 (5.80%)	4 (9.30%)	2 (3.30%)	
Hemodialysis	97 (93.30%)	39 (90.70%)	58 (95.10%)	
Peritoneal dialysis	0	0	0	
Combination	1 (1.00%)	0	1 (1.60%)	
Duration of dialysis (yr) Median (IQR)	1.00 (0.90–2.00)	1.00 (0.50–1.50)	2.00 (1.00–3.00)	<.001
BMI (kg/m^2^) Median (IQR)	20.40 (18.75–21.90)	20.44 (19.41–21.48)	20.39 (18.75–21.92)	.984
Pre-KT Creatinin (mg/dL)	9.36 ± 2.65	8.88 ± 2.58	9.70 ± 2.66	.119
Pre-KT calculated serum creatinine-based eGFR (mL/min/1.73m^2^)	5.73 (4.76–6.79)	6.09 (5.54–7.68)	5.21 (4.58–6.54)	.003
Pre-KT iPTH (pg/mL) Median (IQR)	375.70 (260.70–598.50)	273.00 (206.15–372.10)	489.00 (353.50–845.00)	<.001
Pre-KT Ionized calci (mmol/L) Median (IQR)	1.15 (1.03–1.30)	1.20 (1.07–1.30)	1.11 (1.01–1.30)	.407
Pre-KT Phosphate (mg/dL)	5.79 ± 1.67	5.65 ± 1.74	5.89 ± 1.62	.464
Pre-KT 25OH-Vitamin D(nmol/L)	71.00 ± 14.81	71.09 ± 14.86	70.94 ± 14.90	.960
Pre-KT ALP (U/L) Median (IQR)	96.50 (77.30–125.65)	89.50 (80.15–102.70)	102.00 (76.00–154.00)	.025

ALP = alkaline phosphatase, BMI = body mass index, CKD = chronic kidney disease, ESRD = end-stage renal disease, HPT = hyperparathyroidism, iPTH = intact parathyroid hormone, KT = kidney transplantation.

Longitudinal biochemical changes are presented in Table 2. Linear mixed-effects models demonstrated significant overall changes over time in serum creatinine, eGFR, iPTH, ionized calcium, phosphate, 25(OH) vitamin D, and ALP (all *P* ≤ .001). Bonferroni-adjusted pairwise comparisons showed marked improvement in renal function by month 1, with no significant differences in creatinine or eGFR among months 1, 3, and 6. iPTH decreased significantly from pretransplantation to all posttransplant time points and continued to decline from month 1 to months 3 and 6, whereas no significant difference was observed between months 3 and 6. Ionized calcium increased significantly after transplantation and remained relatively stable thereafter. Phosphate decreased markedly at month 1, followed by a significant increase by months 3 and 6, with no significant difference between months 3 and 6. Compared with pretransplant levels, 25(OH) vitamin D was significantly lower at all posttransplant assessments, whereas ALP was significantly lower at months 3 and 6. Detailed Bonferroni-adjusted pairwise comparisons are provided in Supplementary table 1, Supplemental Digital Content 1.

**Table 2:** Changes of patients’ biochemical characteristics after transplantation.

Characteristics	Pre-KT	Mo 1	Mo 3	Mo 6	Overall *P*-value
Creatinin (mg/dL) Median (IQR)	9.51 (7.05–11.46)	1.23 (1.05–1.60)	1.28 (1.07–1.50)	1.33 (1.07–1.52)	<.001
eGFR (mL/min/1.73m^2^) Median (IQR)	5.73 (4.75–6.79)	67.69 (56.07–78.54)	66.74 (57.14–78.59)	66.22 (57.45–79.11)	<.001
iPTH (pg/mL) Median (IQR)	375.70 (259.00–603.25)	196.00 (139.20–277.98)	172.60 (119.63–233.90)	167.95 (112.53–227.30)	<.001
Ionized calci (mmol/L) Median (IQR)	1.15 (1.03–1.30)	1.23 (1.14–1.30)	1.24 (1.18–1.32)	1.24 (1.17–1.31)	<.001
Phosphate (mg/dL) Mean ± SD	5.79 ± 1.67	2.32 ± 0.88	2.83 ± 0.73	2.83 ± 0.62	<.001
25OH-Vitamin D (nmol/L) Median (IQR)	69.25 (58.90–78.80)	67.05 (53.15–79.33)	61.00 (48.95–76.23)	59.15 (48.98–78.23)	<.001
ALP (U/L) Median (IQR)	96.50 (76.95–126.18)	85.00 (68.48–113.00)	84.25 (61.00–122.75)	76.00 (56.50–111.50)	<.001

Data are presented as mean ± SD or median (IQR), as appropriate. Overall p values were obtained from linear mixed-effects models with time treated as a categorical fixed effect and a patient-specific random intercept to account for within-participant correlation. Post hoc pairwise comparisons were based on estimated marginal means with Bonferroni adjustment and are presented in Supplementary table 1, Supplemental Digital Content 1. Variables with skewed distributions were log-transformed for modeling, while descriptive statistics are presented on their original scales.

ALP = alkaline phosphatase, eGFR = estimated glomerular filtration rate, iPTH = intact parathyroid hormone.

### 3.2. Characteristics of patients with persistent hyperparathyroidism

Among the entire cohort, 58.7% of patients had posttransplant HPT, defined as a PTH level ≥150 pg/mL 6 months post-KT. No significant difference was determined between the 2 groups in terms of age, gender, body mass index, cause of ESRD, or type of dialysis. Compared to those without persistent HPT, patients with persistent HPT had longer durations of CKD from diagnosis (2.00 vs 5.00 years, *P* = .007) and longer durations of dialysis (1.00 vs 2.00 years, *P* < .001). Additionally, patients with posttransplant HPT were more likely to have higher preoperative iPTH and ALP levels (*P* < .001 and *P* = .025, respectively) (Table 1). Using a median threshold of iPTH of 375.70 pg/mL as the cutoff, the probability of HPT recovery was found to be significantly higher in patients with a baseline iPTH level of <375.70 pg/mL (Fig. 2).

**Figure 2.**
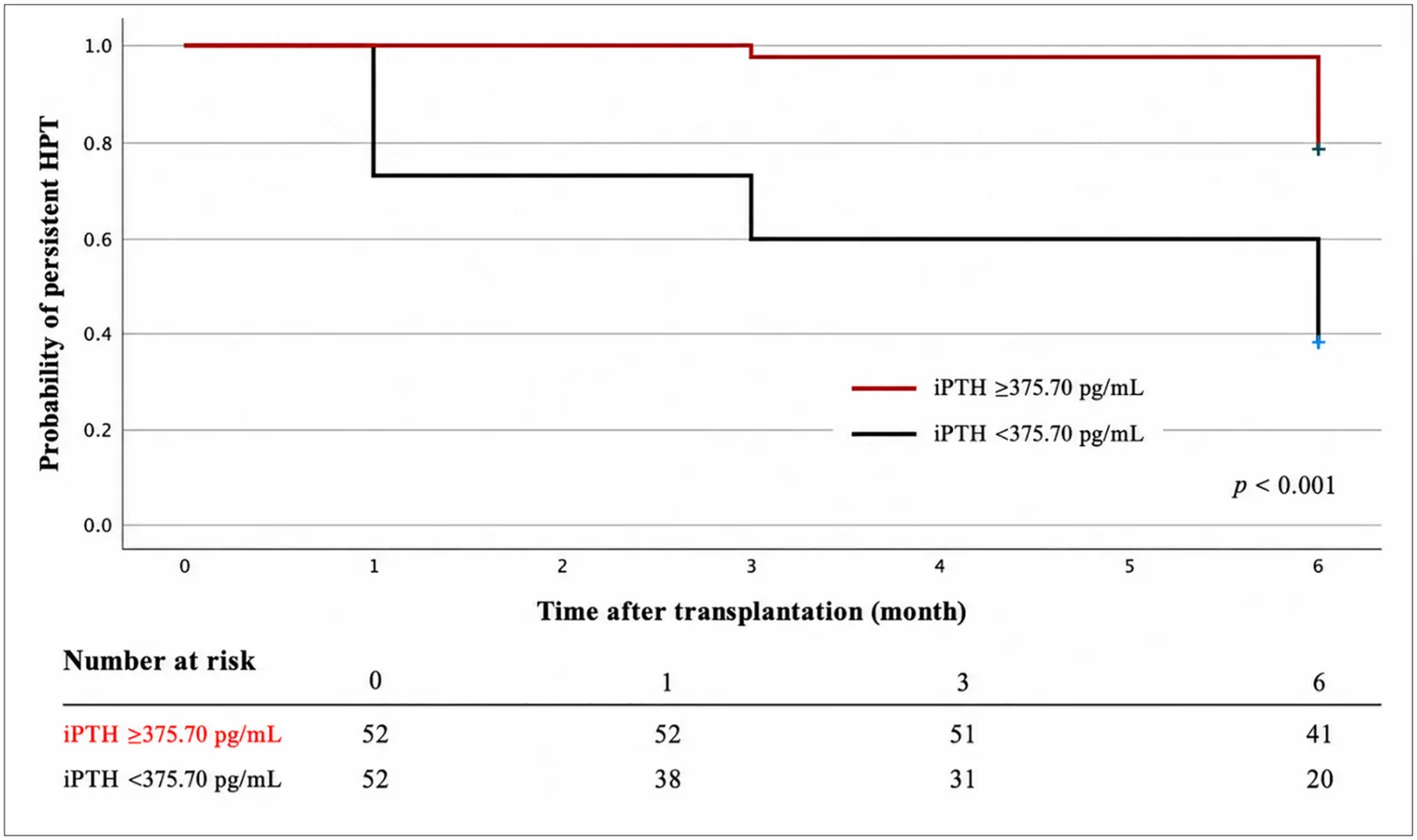
Probability of persistent hyperparathyroidism after kidney transplantation in patients with baseline iPTH levels < 375.70 pg/mL versus ≥ 375.70 pg/mL. HPT = hyperparathyroidism, iPTH = intact parathyroid hormone.

### 3.3. *Factors associated with persistent HPT at 6 months* (Tables 3–4)

Using data on the timing of HPT recovery, univariate logistic regression analysis was initially performed to identify the risk factors for persistent HPT 6 months after transplantation. We found that CKD duration (OR = 1.235, 95% CI = 1.056–1.444), dialysis duration (OR = 1.954, 95% CI = 1.270–3.006), pretransplantation iPTH levels (OR = 1.005, 95% CI = 1.002–1.007), and pretransplantation ALP levels (OR = 1.011, 95% CI = 1.002–1.020) were significantly associated with persistent HPT at 6 months. Additionally, when using median thresholds, CKD duration ≥3 years (OR = 2.295, 95% CI = 1.034–5.092), dialysis duration ≥1 year (OR = 6.107, 95% CI = 2.267–16.453), and pretransplantation iPTH ≥ 375.70 pg/mL (OR = 5.964, 95% CI = 2.501–14.219) were found to be associated with a significantly increased risk of developing persistent HPT (Table 3).

**Table 3:** Univariable logistic regression analysis of factors associated with persistent HPT at 6 months after kidney transplantation.

Variables	Odds ratio, OR	95% CI	*P*-value
Age of recipients	0.998	0.55–1.043	.936
Female	1.298	0.588–2.865	.519
ABO compatibility with donor	0.884	0.312–2.500	.816
CKD duration (yr)	1.235	1.056–1.444	.008
CKD duration ≥3 yr	2.295	1.034–5.092	.041
Cause of ESRD			.276
Glomerulonephritis	1.737	0.626–4.817	
Hypertension	2.605	0.651–10.432	
Other/ Unknown	1		
Dialysis duration (yr)	1.954	1.270–3.006	.002
Dialysis duration ≥1 yr	6.107	2.267–16.453	<.001
BMI (kg/m^2^)	0.991	0.833–1.179	.921
Pre-KT iPTH (pg/mL)	1.005	1.002–1.007	<.001
Pre- KT iPTH ≥375.70 pg/mL	5.964	2.501–14.219	<.001
Pre-KT ionized Ca (mmol/L)	0.900	0.178–4.555	.900
Pre-KT Phosphate (mg/dL)	1.009	0.985–1.033	.460
Pre-KT vitamin D (nmol/L)	0.999	0.973–1.026	.959
Pre-KT ALP (U/L)	1.011	1.002–1.020	.019

ALP = alkaline phosphatase, BMI = body mass index, CKD = chronic kidney disease, ESRD = end-stage renal disease, HPT = hyperparathyroidism, iPTH = intact parathyroid hormone, KT = kidney transplantation.

**Table 4:** Multivariable logistic regression analysis of factors associated with persistent HPT at 6 months after kidney transplantation.

Model 1:	Odd ratio	95% CI	*P*-value
CKD duration (yr)	1.064	0.896–1.263	.481
Dialysis duration (yr)	1.302	0.764–2.217	.331
Pre- KT iPTH (pg/mL)	1.004	1.001–1.006	.009
Pre-KT ALP (U/L)	1.006	0.994–1.017	.335
**Model 2:**	**Odd ratio**	**95% CI**	***P*-value**
CKD duration ≥3 yr	1.224	0.481–3.112	.672
Dialysis duration ≥1 yr	3.886	1.330–11.354	.013
Pre- KT iPTH ≥375.70 pg/mL	3.809	1.463–9.917	.006
Pre-KT ALP (U/L)	1.005	0.996–1.014	.258

Model 1 included CKD duration, dialysis duration, pretransplant iPTH, and ALP as continuous variables. Model 2 included categorized CKD duration (≥3 years), dialysis duration (≥1 year), and pretransplant iPTH (≥375.70 pg/mL), with ALP retained as a continuous variable.

ALP = alkaline phosphatase, CKD = chronic kidney disease, HPT = hyperparathyroidism, iPTH = intact parathyroid hormone, KT = kidney transplantation.

Multivariate logistic regression analyses using 2 different models showed that pre- transplantation iPTH levels were an independently associated with persistent HPT at 6 months in both models (Table 4). Each multivariable model included 4 predictor parameters for 61 persistent-HPT events, corresponding to approximately 15.3 events per parameter. No evidence of problematic multicollinearity was observed. VIFs ranged from 1.300 to 3.149 in Model 1 and from 1.106 to 1.224 in Model 2, with corresponding tolerance values of 0.318 to 0.769 and 0.817 to 0.904, respectively. The Hosmer–Lemeshow tests showed no evidence of poor model fit for either Model 1 (*χ*^*2*^ = 4.972, *P* = 761) or Model 2 (*χ*^*2*^ = 9.213, *P* = .325). Nagelkerke *R*^*2*^ values were 0.348 and 0.331, respectively. Model 1 showed good discrimination, with an AUC of 0.804 (95% CI, 0.720–0.889), whereas Model 2 had an AUC of 0.768 (95% CI, 0.677–0.859). Detailed model diagnostics are provided in Supplementary Table 2, Supplemental Digital Content 2. Specifically, for every 1 pg/mL increase in iPTH levels, the risk of developing persistent HPT increases by 0.4%.

In the multivariable analysis using continuous variables, pretransplant iPTH remained independently associated with persistent HPT (adjusted OR 1.004 per 1-pg/mL increase, 95% CI 1.001–1.006; *P* = .009), whereas dialysis duration was not statistically significant (adjusted OR 1.302 per year, 95% CI 0.764–2.217; *P* = .331). In the model using categorized variables, both pre-transplant iPTH ≥375.70 pg/mL (adjusted OR 3.809, 95% CI 1.463–9.917; *P* = .006) and dialysis duration ≥1 year (adjusted OR 3.886, 95% CI 1.330–11.354; *P* = .013) were independently associated with persistent HPT. Thus, the association between dialysis duration and persistent HPT was dependent on how dialysis exposure was modeled.

## 4. Discussion

Even with successful KT, the persistence of elevated iPTH levels is a substantial clinical challenge, as it is associated with a wide range of adverse outcomes, including an increased risk of fracture, cardiovascular events, allograft loss, and even mortality. ^[9–12]^ This study provides valuable insights into persistent HPT following KT in a cohort of patients at Cho Ray Hospital, a tertiary hospital in the south of Vietnam. The reported prevalence of persistent HPT varies substantially among studies because no universally accepted diagnostic criterion currently exists. Previous investigations have used different PTH thresholds and assessment time points, making direct comparison difficult. We adopted an iPTH threshold of ≥150 pg/mL at 6 months because this cutoff was previously shown to provide high specificity for predicting persistent HPT at 1 year after KT. Our findings indicate a significant prevalence of persistent HPT, affecting 58.65% of kidney transplant recipients at 6 months posttransplantation. This rate is comparable to figures recently reported in the literature; for example, Sutton et al found that 61.7% of kidney recipients had persistent HPT 1 year after KT.^[5]^ While our prevalence rate is consistent with recent studies, some research has presented different figures, with rates for persistent HPT after KT ranging from 10% to 50%.^[1,2,13]^ These discrepancies can largely be attributed to differences in the definition of persistent HPT and the duration of follow-up. For instance, Evenepoel et al defined persistent HPT as an iPTH level ≥2.5 times the upper normal limit, whereas our study used a threshold of 150 pg/mL at 6 months. Differences in the time points used for assessment also influence the reported prevalence of HPT. This variability in reported prevalence rates highlights the need for a standardized definition of persistent HPT and reinforces the value of this study in providing a specific prevalence rate at a standardized 6-month time point. The high prevalence observed in our cohort emphasizes that persistent HPT represents a common persistent complication of CKD after KT rather than a consequence of successful transplantation, highlighting the need for careful monitoring and appropriate management.

This study demonstrates the physiological recovery of renal and metabolic functions after successful KT. Renal function improved markedly within the first month and remained relatively stable thereafter. In contrast, iPTH showed a more gradual decline, with significant reductions continuing through month 3 but no further significant change between months 3 and 6.This pattern suggests that recovery of parathyroid function may lag behind restoration of graft function. Although renal function and phosphate homeostasis are normalized, the resolution of HPT is incomplete, and ionized calcium levels remain elevated. Median iPTH levels fall significantly, from 375.70 pg/mL pre-KT to 167.95 pg/mL after 6 months.

However, this reduction does not lead to a complete normalization of parathyroid function for the majority of patients. The persistence of elevated iPTH levels suggests that HPT pathophysiology extends beyond simple renal dysfunction. In CKD, prolonged stimulation of the parathyroid glands causes hyperplasia and nodular remodeling, reducing responsiveness to negative calcium and vitamin D feedback. Additionally, low vitamin D and elevated FGF23 levels, and ongoing hyperphosphatemia further stimulate PTH secretion.^[14–17]^ The current study reinforces the idea that the severity of preexisting secondary HPT significantly influences posttransplant HPT outcomes.

In our cohort, advanced therapeutic agents for SHPT, such as calcimimetics and non-calcium-based phosphate binders, were not widely available due to limited supply in our country, high cost, and absence of insurance reimbursement, thereby limiting optimal treatment opportunities for ESRD patients. Furthermore, prior to being referred to our center for KT, these ESRD patients had been managed across multiple different healthcare facilities. This fragmented pre-transplant care, combined with resource limitations, resulted in suboptimal medical management and inconsistent tracking of their SHPT treatment regimens. With respect to parathyroid imaging, pretransplant cervical ultrasonography was routinely performed as part of the evaluation of secondary HPT. The presence of parathyroid nodules was recorded and has now been incorporated into the revised manuscript as a baseline characteristic. However, because only a limited number of patients demonstrated nodular disease, this variable did not materially alter the multivariable analysis.

This study identified several baseline factors associated with persistent HPT at 6 months after KT. Patients with persistent HPT exhibit significantly higher baseline serum iPTH levels and ALP, alongside a longer duration of CKD and longer dialysis duration. Higher pretransplant iPTH showed a consistent independent association with persistent HPT across both multivariable model specifications. In contrast, the association with dialysis duration was model-dependent. Dialysis duration ≥1 year was independently associated with persistent HPT in the categorized model, whereas dialysis duration modeled as a continuous variable was not statistically significant after adjustment. These findings support existing research showing that high pre-KT iPTH levels and prolonged dialysis are crucial associated factors for developing persistent HPT.^[1,2,6,7]^ The identification of pretransplant iPTH levels and dialysis duration as independent associated factors provides a clear roadmap for managing high-risk patients. These findings suggest that patients with high pre-KT iPTH levels and a history of prolonged dialysis require close monitoring and possibly therapeutic intervention requiring further evaluation.

The strength of this study comes from its prospective cohort design, allowing for the sequential evaluation of HPT and biochemical metabolic parameters in a consistent manner. Detailed biochemical assessments at multiple time points provide a comprehensive picture of the posttransplant trajectory.

However, this study has some limitations. First, the study was conducted at a single center (Cho Ray Hospital, Vietnam), which may limit the generalizability of the findings to other populations or healthcare settings. Second, the sample size of 104 participants, while substantial for a single-center study, is relatively modest compared to large-scale multicenter studies. Although model complexity was restricted relative to the number of observed outcome events, the relatively small sample size may still have resulted in imprecise or unstable estimates, particularly for categorized predictors with relatively wide confidence intervals. No formal prediction model development or internal/external validation was undertaken. Therefore, the multivariable findings should be regarded as exploratory associations requiring confirmation in larger independent cohorts.Third, detailed information regarding pretransplant medical treatment for secondary HPT, including calcimimetic therapy, vitamin D analogues, phosphate binders, and medication dosage, was not available, precluding assessment of their potential influence on posttransplant parathyroid recovery. Finally, the 6-month follow-up period should be considered an early assessment of posttransplant parathyroid recovery rather than a definitive evaluation of long-term persistent HPT. Although previous studies have demonstrated that parathyroid function may continue to improve during the first year after transplantation, early identification of patients with persistent elevation of PTH remains clinically relevant because these patients represent a high-risk group for ongoing disturbances in mineral metabolism and transplant-related complications. Therefore, the factors identified in our study should be interpreted as factors associated with persistent HPT at the 6-month assessment rather than as established causal or long-term predictive determinants. Future prospective studies with follow-up extending to at least 12 months are warranted to determine whether these associations persist over longer-term follow-up.

## 5. Conclusion

Persistent HPT remains a prevalent complication after successful KT and is significantly influenced by pretransplant iPTH levels and dialysis duration ≥1 year. While improvements in biochemical parameters generally occur post-KT, a substantial proportion of patients continue to experience HPT. These findings underscore the importance of monitoring and applying appropriate strategies for early prevention in kidney transplant recipients in order to mitigate potential long-term complications. Further multicenter studies with longer follow-up periods are warranted to fully elucidate the long-term outcomes and optimize therapeutic approaches for persistent HPT after KT.

## Author contributions

**Data curation:** Kim-Ngan Huynh Lam, Hen Huu Phan, Sam Minh Thai.

**Formal analysis:** Kim-Ngan Huynh Lam, Minh Duc Do.

**Investigation:** Kim-Ngan Huynh Lam.

**Conceptualization:** Hen Huu Phan, Sam Minh Thai.

**Validation:** Hen Huu Phan, Sam Minh Thai, Minh Duc Do.

**Supervision:** Sam Minh Thai.

**Methodology:** Minh Duc Do.

**Writing – original draft:** Kim-Ngan Huynh Lam, Hen Huu Phan, Minh Duc Do.

**Writing – review & editing:** Minh Duc Do.




