# Supplementary material for: Persistent hyperparathyroidism and its predictive factors in end-stage renal disease patients after kidney transplantation: A prospective study
Source: Medicine (Baltimore). 2026 Oct 16;105(42):e51216. doi: 10.1097/MD.0000000000051216 (PMC13681121; doi:10.1097/MD.0000000000051216)
Supplement: Supplementary file 1 [file medi-105-e51216-s001.docx]

Supplementary table 1. Bonferroni-adjusted pairwise comparisons of longitudinal biochemical measurements

| **Variable** | **Comparison** | **Mean difference/ model contrast** | **95%CI** | **Adjusted p** |
| --- | --- | --- | --- | --- |
| Serum Creatinine | Pre-KT vs M1 | 1.957 | 1.853 to 2.061 | <0.001 |
|  | Pre-KT vs M3 | 1.953 | 1.858 to 2.047 | <0.001 |
|  | Pre-KT vs M6 | 1.938 | 1.863 to 2.013 | <0.001 |
|  | M1 vs M3 | -0.004 | -0.079 to 0.071 | 1.000 |
|  | M1 vs M6 | -0.019 | -0.113 to 0.076 | 1.000 |
|  | M3 vs M6 | -0.015 | -0.090 to 0.061 | 1.000 |
| eGFR | Pre-KT vs M1 | -2.416 | -2.521 to -2.311 | <0.001 |
|  | Pre-KT vs M3 | -2.412 | -2.508 to -2.316 | <0.001 |
|  | Pre-KT vs M6 | -2.402 | -2.480 to -2.325 | <0.001 |
|  | M1 vs M3 | 0.004 | -0.073 to 0.081 | 1.000 |
|  | M1 vs M6 | 0.014 | -0.082 to 0.109 | 1.000 |
|  | M3 vs M6 | 0.010 | -0.068 to 0.087 | 1.000 |
| iPTH | Pre-KT vs M1 | 0.726 | 0.605 to 0.847 | <0.001 |
|  | Pre-KT vs M3 | 0.887 | 0.752 to 1.023 | <0.001 |
|  | Pre-KT vs M6 | 0.893 | 0.754 to 1.033 | <0.001 |
|  | M1 vs M3 | 0.161 | 0.041 to 0.282 | 0.003 |
|  | M1 vs M6 | 0.167 | 0.032 to 0.303 | 0.007 |
|  | M3 vs M6 | 0.006 | -0.115 to 0.127 | 1.000 |
| Ionized calci | Pre-KT vs M1 | -0.062 | -0.109 to -0.014 | 0.004 |
|  | Pre-KT vs M3 | -0.087 | -0.135 to -0.040 | <0.001 |
|  | Pre-KT vs M6 | -0.088 | -0.134 to -0.042 | <0.001 |
|  | M1 vs M3 | -0.026 | -0.072 to 0.021 | 0.843 |
|  | M1 vs M6 | -0.026 | -0.074 to 0.021 | 0.857 |
|  | M3 vs M6 | -0.001 | -0.047 to 0.046 | 1.000 |
| Phosphate | Pre-KT vs M1 | 3.478 | 3.090 to 3.865 | <0.001 |
|  | Pre-KT vs M3 | 2.961 | 2.579 to 3.342 | <0.001 |
|  | Pre-KT vs M6 | 2.959 | 2.609 to 3.309 | <0.001 |
|  | M1 vs M3 | -0.517 | -0.867 to -0.166 | <0.001 |
|  | M1 vs M6 | -0.519 | -0.900 to -0.137 | 0.002 |
|  | M3 vs M6 | -0.002 | -0.352 to 0.349 | 1.000 |
| 25OH vitamin D | Pre-KT vs M1 | 0.085 | 0.006 to 0.165 | 0.027 |
|  | Pre-KT vs M3 | 0.127 | 0.030 to 0.225 | 0.004 |
|  | Pre-KT vs M6 | 0.149 | 0.042 to 0.255 | 0.001 |
|  | M1 vs M3 | 0.042 | -0.037 to 0.121 | 0.969 |
|  | M1 vs M6 | 0.063 | -0.035 to 0.161 | 0.539 |
|  | M3 vs M6 | 0.021 | -0.059 to 0.102 | 1.000 |
| ALP | Pre-KT vs M1 | 0.120 | 0.000 to 0.241 | 0.051 |
|  | Pre-KT vs M3 | 0.167 | 0.051 to 0.284 | 0.001 |
|  | Pre-KT vs M6 | 0.233 | 0.130 to 0.336 | <0.001 |
|  | M1 vs M3 | 0.047 | -0.056 to 0.150 | 1.000 |
|  | M1 vs M6 | 0.113 | -0.004 to 0.229 | 0.064 |
|  | M3 vs M6 | 0.066 | -0.037 to 0.169 | 0.542 |
