# Supplementary material for: Persistent hyperparathyroidism and its predictive factors in end-stage renal disease patients after kidney transplantation: A prospective study
Source: Medicine (Baltimore). 2026 Oct 16;105(42):e51216. doi: 10.1097/MD.0000000000051216 (PMC13681121; doi:10.1097/MD.0000000000051216)
Supplement: Supplementary file 2 [file medi-105-e51216-s002.docx]

Supplementary table 2. Diagnostic performance and stability of the multivariable logistic regression models

| **Model diagnostic** | **Model 1:** | **Model 2:** |
| --- | --- | --- |
| Number of participants | 104 | 104 |
| Persistent-HPT events | 61 | 61 |
| Parameters | 4 | 4 |
| Events/parameter | 15.3 | 15.3 |
| Nagelkerke R² | 0.348 | 0.331 |
| Hosmer–Lemeshow χ² | 4.972 | 9.213 |
| Hosmer–Lemeshow P | 0.761 | 0.325 |
| AUC (95% CI) | 0.804  (0.720 – 0.889) | 0.768  (0.677 – 0.859) |
| VIF range | 1.300 – 3.149 | 1.106 – 1.224 |
| Tolerance range | 0.318 – 0.769 | 0.817 – 0.904 |

Model 1 included CKD duration, dialysis duration, pre-transplant iPTH, and ALP as continuous variables. Model 2 included categorized CKD duration (≥3 years), dialysis duration (≥1 year), and pre-transplant iPTH (≥375.70 pg/mL), with ALP retained as a continuous variable. AUC, area under the receiver operating characteristic curve; VIF, variance inflation factor.
